# Preliminary Identification of the Aerobic Cervicovaginal Microbiota in Mexican Women With Cervical Cancer as the First Step Towards Metagenomic Studies

**DOI:** 10.3389/fcimb.2022.838491

**Published:** 2022-02-02

**Authors:** Gauddy Lizeth Manzanares-Leal, Jaime Alberto Coronel-Martínez, Miguel Rodríguez-Morales, Iván Rangel-Cuevas, Lilia Patricia Bustamante-Montes, Horacio Sandoval-Trujillo, Ninfa Ramírez-Durán

**Affiliations:** ^1^ Laboratory of Medical and Environmental Microbiology, School of Medicine, Universidad Autonoma del Estado de Mexico, Toluca, Mexico; ^2^ Departments of Clinical Research and Medical Oncology, Instituto Nacional de Cancerologia, Mexico City, Mexico; ^3^ Genomics Laboratory, Instituto Nacional de Cancerologia, Mexico City, Mexico; ^4^ Gynecology Department, Maternal and Child Hospital, Instituto de Seguridad Social del Estado de México y Municipios, Toluca, Mexico; ^5^ Deanship of Health Sciences, Universidad Autonoma de Guadalajara, Guadalajara, Jalisco, Mexico; ^6^ Department of Biological Systems, Universidad Autonoma Metropolitana-Xochimilco, Mexico City, Mexico

**Keywords:** cervical cancer, aerobic vaginitis, aerobic bacteria, human microbiome, cervicovaginal microbiome

## Abstract

Cervical cancer (CC) is considered a public health problem. Recent studies have evaluated the possible relationship between the cervicovaginal microbiome and gynecologic cancer but have not studied the relationship between aerobic bacterial communities and neoplasia. The study aimed to identify the cultivable aerobic bacterial microbiota in women with cervical cancer as a preliminary approach to the metagenomic study of the cervicovaginal microbiome associated with cervical cancer in Mexican women. An observational cross-sectional study was conducted, including 120 women aged 21-71 years, divided into two study groups, women with locally advanced CC (n=60) and women without CC (n=60). Sociodemographic, gynecological-obstetric, sexual, and habit data were collected. Cervicovaginal samples were collected by swabbing, from which standard microbiological methods obtained culturable bacteria. The strains were genetically characterized by PCR-RFLP of the 16S *r*RNA gene and subsequently identified by sequencing the same gene. Variables regularly reported as risk factors for the disease were found in women with CC. Differences were found in the prevalence and number of species isolated in each study group. Bacteria commonly reported in women with aerobic vaginitis were identified. There were 12 species in women with CC, mainly *Corynebacterium* spp. and *Staphylococcus* spp.; we found 13 bacterial species in the group without cancer, mainly *Enterococcus* spp. and *Escherichia* spp. The advanced stages presented a more significant number of isolates and species. This study provided a preliminary test for cervicovaginal metagenomic analysis, demonstrating the presence of aerobic cervicovaginal dysbiosis in women with CC and the need for more in-depth studies.

## Introduction

Cervical cancer (CC) is one of the significant public health problems in the world. Globally, it represents the seventh most common cancer in humans and the fourth most common cancer in women, but it reaches second place in incidence and mortality in settings with a low human development index (Bray et al., 2018). In Mexico, this condition is aggravated because the diagnosis mainly occurs in locally advanced stages (IB-2 to IV-A), placing the pathology as the second leading cause of cancer death in women (Baezconde-Garbanati et al., 2019; Martínez-Rodríguez et al., 2021).

Recent studies have evaluated the relationship between the cervicovaginal microbiome and gynecological cancer to elucidate the involvement of bacterial communities in the establishment, progression, or cure of the disease. Dysbiosis due to the presence of bacterial vaginosis is the most studied entity, found to have a possible association with the induction of local inflammation and lactic acid depletion (Anahtar et al., 2015; Łaniewski et al., 2018), persistent high-risk Human Papillomavirus (HPV-Hr) infection (Jørgensen et al., 2009; Gao et al., 2013; Piyathilake et al., 2016; Ilhan et al., 2019; Norenhag et al., 2020; Wei et al., 2020), Cervical Intraepithelial Neoplasia (CIN) (Gillet et al., 2012; Huang et al., 2020) and the establishment of cervical cancer (Kwasniewski et al., 2018; Huang et al., 2020; Kang et al., 2021).

Among the main bacterial genera reported so far concerning CC are *Gardnerella, Prevotella, Dialister, Slackia, Actinomyces, Porphyromonas, Peptoniphilus, Anaerococcus, Peptostreptococcus, Streptococcus, Ureaplasma, Megasphaera, Mycoplasma, Sneathia, Mobiluncus, Fusobacterium,* and *Atopobium* (Anahtar et al., 2015; Di Paola et al., 2017; Wei et al., 2020), all of which are facultative anaerobic or strict anaerobic bacteria.

In 2002, Donders et al. (2002) reported the existence of a pathological entity called aerobic vaginitis, characterized by a high abundance and diversity of aerobic, enteric bacteria and inflammatory symptoms. In 2019, Wang et al. (2020) evaluated the bacterial profiles of aerobic vaginitis by both culture-dependent and molecular methods. They found no single profile to describe the vaginal bacteriome in patients with aerobic vaginitis. In 2021, Plisko et al. (2021) published a paper whose objective was to analyze the association between the vaginal microbiota of women with aerobic vaginitis and the presence of cervical intraepithelial neoplasia. In this study, aerobic microbiota was detected by wet-mount microscopy. The authors concluded that there might be a relationship between aerobic dysbiotic cervicovaginal microbiota and the development of cervical intraepithelial lesions.

Currently, to our knowledge, the aerobic microbiota present in women with established cervical cancer has not been evaluated. Therefore, this study aimed to identify the culturable aerobic bacterial microbiota in women with locally advanced cervical cancer as an approach before the metagenomic study of the cervicovaginal microbiome associated with cervical cancer in Mexican women.

## Method

### Study Design and Ethical Aspects

A cross-sectional, descriptive, observational study was carried out. Cervicovaginal fluid samples were collected by swabbing. Sample collection was developed from February 2016 to February 2018. The study protocol was approved by the National Cancer Institute of Mexico (INCan) with the code 016/11/ICI-ICI-CEI/1016. All procedures were carried out under proper bioethical standards. All women who decided to participate in the study signed the corresponding informed consent form.

### Study Groups

Two study groups were included, according to the following characteristics: Study group 1 (CC): women older than 18 years, newly diagnosed with locally advanced cervical cancer, according to the International Federation of Gynecology and Obstetrics (FIGO) staging system for cervical cancer (Saleh et al., 2020), and histologically confirmed (n=60); Study group 2 (Non-CC): women older than 18 years, without cervical cancer, confirmed with negative cervical cytology, companions (family members or neighbors) of women in study group 1 or who attended screening appointments (n=60).

The following exclusion criteria were applied: pregnant women, women who used systemic and local antimicrobials and antifungals 30 days before sampling, women who had sexual intercourse 48 hours before sampling, women who used vaginal douching, and who were menstruating on the day of sampling.

### Study Population Characteristics

A questionnaire was applied to all participants to obtain sociodemographic data (age, education level, and marital status); gynecological-obstetric data (number of vaginal deliveries); sexual data (start of active sexual life, number of sexual partners, and use of hormonal contraceptive methods); habits (smoking); and disease data collected from clinical histories (clinical stage of the disease, histological type of tumor).

### Sample Processing

Cervicovaginal fluid samples were inoculated in Petri dishes with Brain Heart Infusion (BHI) agar (Becton Dickinson, Cat. No. 214700) and in Petri dishes with blood agar (Becton Dickinson, Cat. No. 220150). The Petri dishes were incubated at 37°C for 48 hr under aerobic conditions. Once the incubation time had elapsed, the predominant colonies were isolated and purified.

### DNA Extraction

The isolated and purified colonies were inoculated in BHI broth to obtain biomass, incubated at 37°C for 48 hr. Biomass recovery was performed by centrifugation at 10000 rpm for 10 minutes. Subsequently, the manufacturer’s instructions performed DNA extraction using the Promega Wizard^®^ Genomic kit (Cat. No. A1120).

### Amplification of the 16S *r*RNA Gene

The 16S *r*RNA gene was amplified with the DNA obtained by Polymerase Chain Reaction (PCR). Taq DNA Polymerase (My Taq, Bioline. Cat. No. BIO-21105) was used and the following nucleotide sequences were used as universal primers: 27f: 5’- AGAGTTTGATCMTGGCTCAG-3’; 1492r: 5’- TACGGYTACCTTGTTACGACTT-3’; 1492r: 5’- TACGGYTACCTTGTTACGACTT-3’. The conditions used in thermal cycling for gene amplification were as follows: Initial denaturation, a 5-minute cycle at 94°C, followed by 29 cycles of denaturation for 1 minute at 94°C, annealing 30 seconds at 59°C, extension 1minute at 72°C. The final extension was 10 minutes at 72°C.

The amplified fragments were observed by 1% agarose gel electrophoresis (Conda Pronadisa, cat. no. 8100.10), stained with ethidium bromide (SIGMA cat. no. E7637-1G). The electrophoresis conditions were: 120 V for 40 min, in TAE buffer (TAE buffer 1X Invitrogen, Cat. No. 24710-030) as a running buffer.

### Genomic Characterization

The amplicons of the 16S *r*RNA gene of the isolated strains were used to type the strains obtained to determine the genotypic differences of the isolates. For this purpose, PCR-RFLP ribotyping of the 16S *r*RNA gene with MSPI (Promega, Cat. No. R6401) and RSAI (Promega, Cat. No. R6371) enzymes were used. For both enzymes, the restriction reaction was carried out for 1 h at 37°C, inactivated by heating 15 min at 72°C, according to the manufacturer’s instructions.

Restriction products were observed by 1.5% agarose gel electrophoresis (Conda Pronadisa, Cat. No. 8100.10), stained with ethidium bromide (SIGMA Cat. No. E7637-1G), under the following conditions: 120V for 80 minutes with TAE buffer (TAE buffer 1X Invitrogen, Cat. No. 24710-030) and 1 kb DNA molecular weight marker (Thermo Scientific, Cat. No. 5M0311).

The restriction patterns generated were analyzed according to the number of bands and size concerning the molecular weight marker used. A “ribotype” was defined as a group of strains with identical enzymatic restriction profiles

### Genetic Identification

For genetic identification, between 15% and 50% of the strains from the ribotypes formed were selected. Once the representative strains were chosen, a second amplification of the 16S *r*RNA gene was performed by PCR, following the methodology described above. The amplification products were purified with the Amicon Ultra filter^®^ kit (Millipore, Cat. No. UFC500308) and sent to the Macrogen USA sequencing service (Macrogen Sequenciation Service, Maryland, USA).

The sequences were analyzed and corrected with the following programs: ChromasPro version 2.6.4 (Technelysium Pty Ltd, South Brisbane, Australia) and BioEdit version 5.0.9 (Hall, 1999). Consensus sequences were constructed and compared with sequences deposited in the GenBank of the National Center for Biotechnology Information (NCBI) using the BLAST (Basic Local Alignment Search Tool) program (Altschul et al., 1990) as well as in the EzBiocloud public database (Yoon et al., 2017).

### Statistical Analysis

Sociodemographic, gynecological-obstetric, sexual, habit, and disease characteristics and the number of isolated strains were analyzed using descriptive statistics. The Student’s t-test compared the mean age of the participants. The Chi2 test was used to compare the proportions of the remaining variables and the number of isolates per culture medium. The Cochran-Mantel-Haenszel test was applied to rule out the possibility of sociodemographic variables acting as confounding variables in the presence or absence of isolated bacteria. For women with CC, analysis of the proportion of strains obtained by subgroup, stratified by parametrial and non-parametrial invasion, and tumor type was performed using Fisher’s exact test. A significance level of p<0.05 was considered. Stata version 15 was used for the analysis of all data evaluated.

## Results

### Sociodemographic, Gyneco-Obstetric, Sexual, and Habit Characteristics

Regarding the characteristics of the study population, the groups did not differ in age. Still, statistically significant differences were found in the level of education (p=0.01), vaginal deliveries (p<0.01), beginning of active sexual life (p<0.01), and use of contraceptive methods (p=0.02) (**Table 1**). For the women with CC, the clinical stage of the disease most frequently was IIB (50%), and squamous cell carcinoma accounted for 85% of the cases (**Table 1**). Statistically, no variable in this item acted as a confounder.

**Table 1:** Characteristics of the study population.

Characteristic	Study group 1 (CC) (n=60)	Study group 2 (Non-CC) (n=60)	Significance
	Sociodemographic characteristics	
Age	Mean (range, S.D.)	Mean (range, S.D.)	
	46 (22-68, 11.9)	45 (21-71, 11.7)	0.57
Education level	Frequency (%)	Frequency (%)	
None	12 (20.0)	5 (8.3)	0.01*
Elementary	20 (33.3)	13 (21.6)	
Secondary	19 (31.6)	19 (31.6)	
High school	8 (13.3)	15 (25.0)	
University and postgraduate	3 (5.0)	8 (13.3)	
Marital Status			
Married/free union	31 (51.6)	36 (60.0)	0.16
Widowed	6 (10.0)	3 (5.0)	
Single	23 (38.3)	21 (35.0)	
	Obstetrical and gynecological characteristics	
Deliveries	Frequency (%)	Frequency (%)	
≤ 3	38 (63.3)	56 (93.3)	<0.01*
>3	22 (36.6)	4 (6.6)	
	Sexual characteristics	
Beginning of active sexual life	Frequency (%)	Frequency (%)	
≤ 18 years	38 (63.3)	27 (45.0)	<0.01*
>18 years	22 (36.6)	33 (55.0)	
Number of sexual partners			
≤ 3	53 (88.3)	49 (81.6)	0.44
>3	7 (11.6)	11 (18.3)	
Contraceptive method use			
None	31 (51.6)	28 (46.6)	0.02*
Hormonal	19 (31.6)	10 (16.6)	
Non- hormonal	10 (16.6)	22 (36.6)	
	Habits	
Smoking habit	Frequency (%)	Frequency (%)	
Yes	11 (18.3)	18 (30.0)	0.66
No	49 (81.6)	42 (70.0)	
	Disease	
Clinical stage	Frequency (%)	Frequency (%)	
IB2	9 (15.0)	N/A	N/A
IIA	3 (5.0)	N/A	N/A
IIA1	1 (1.6)	N/A	N/A
IIA2	2 (3.3)	N/A	N/A
IIB	30 (50.0)	N/A	N/A
IIIA	2 (3.3)	N/A	N/A
IIIB	8 (13.3)	N/A	N/A
IVA	5 (8.3)	N/A	N/A
Tumor histological type			
Squamous cell carcinoma	51 (85.0)	N/A	N/A
Adenocarcinoma	9 (15.0)	N/A	N/A

S.D, Standard deviation, %, percentage, N/A, not applicable. *Significance level p < 0.05,Student’s t-test or chi2 test.

### Strains Isolated

From study group 1 (CC), 116 strains were isolated; 52 on BHI agar (44.8%) and 64 on blood agar (55.2%). In group 2 (Non-CC), 67 strains were isolated; 21 on BHI agar (31.3%) and 46 on blood agar (68.7%). It can be observed that the group of women with CC presented a superior number of isolated strains; however, when comparing the proportions by type of culture medium, this result was not statistically significant (p=0.07).

### Genomic Characterization

The 16S rRNA gene of the isolated strains was characterized using restriction enzymes. The gene fragments generated by the enzymes could be visualized and adequately separated by agarose gel electrophoresis; this generated bands that varied in number, size, and arrangement, which showed that the strains were genetically distinct and allowed their classification by grouping patterns of similar bands or ribotypes. Thus, 12 ribotypes were found in the group of women with CC, and 13 ribotypes were found in Study Group 2 (Non-CC) (**Table 2**). Using two restriction enzymes (MSPI and RSAI), we corroborated that the strains clustered as described, and the groups were genetically distinct.

**Table 2:** Molecularly identified strains, divided by ribotype and study group.

Ribotype No	Group 1 (CC)					Group 2 (Non-CC)				
	Strains by ribotype	Strains identified by 16S rRNA (%)	Species identified	BLAST%	EzBiocloud%	Strains by ribotype	Strains identified by 16S rRNA (%)	Species identified	BLAST%	EzBiocloud%
1	35	9 (25.7)	*Staphylococcus epidermidis*	98-99	98-99	17	8 (47.05)	*Enterococcus faecalis*	99-100	97-99
2	24	9 (37.5)	*Corynebacterium amycolatum*	97-100	97-99	13	6 (46.15)	*Escherichia fergusonii*	99-100	99
3	14	5 (35.7)	*Enterococcus faecalis*	97-100	98-99	6	2 (33.3)	*Facklamia hominis*	97-99	99
4	8	4 (50.0)	*Escherichia fergusonii*	97-100	99	5	3 (60.0)	*Staphylococcus epidermidis*	97-98	99-100
5	6	3 (50.0)	*Corynebacterium jeikeium*	98-100	97-99	5	2 (40.0)	*Staphylococcus auricularis*	97-99	98
6	6	3 (50.0)	*Streptococcus agalactiae*	98-100	98	4	2 (50.0)	*Corynebacterium amycolatum*	99-100	98
7	6	1 (16.6)	*Bacillus safensis*	98	99	4	1 (25.0)	*Brevibacterium masiliense*	99	99
8	5	1 (20.0)	*Lactobacillus rhamnosus*	97	100	3	1 (33.3)	*Streptococcus agalactiae*	99	99
9	4	2 (50.0)	*Streptococcus urinalis*	100	97-99	3	1 (33.3)	*Klebsiella oxytoca*	99	99
10	4	1 (25.0)	*Bacillus malikii*	97	99	2	1 (50.0)	*Staphylococcus pasteuri*	100	99
11	3	1 (33.3)	*Escherichia coli*	97	99	2	1 (50.0)	Paenibacillus urinalis	100	99
12	1	1 (100)	*Corynebacterium striatum*	97	99	2	1 (50.0)	*Staphylococcus capitis*	98	99
13	–	–	*-*	–	–	1	1 (100)	*Pseudocitrobacter faecalis*	100	98
Total	116	40 (34.5)	12 species			67	30 (44.7)	13 species		

### Genetic Identification

Identification by 16S *r*RNA gene sequencing was performed on 40 strains representative of the 12 ribotypes of study group 1 (CC). Consensus sequences between 1311bp and 1472bp were obtained, with similarity percentages between 97% and 100%. Seven genera and 12 bacterial species were identified. The species corresponded to each of the ribotypes established in this study group (**Table 2**).

From Study Group 2 (Non-CC), 30 strains of 13 ribotypes were chosen. Consensus sequences between 1391bp and 1436bp were obtained with 97% and 100% similarity percentages. 10 genera and 13 bacterial species were identified. The species correspond to the ribotypes established in this group (**Table 2**).

The obtained sequences were deposited in the GenBank of the National Center for Biotechnology Information with access numbers MH108987−MH109026 (Group 1) and MH158254-MH158283 (Group 2).

Both women with CC and those without the disease presented mostly facultative aerobic and enteric bacteria. *Staphylococcus epidermidis*, *Streptococcus agalactiae, Enterococcus faecalis, Escherichia fergusonii*, and *Corynebacterium amycolatum* species were identified in both study groups, with percentages between 5% and 30% of presence in the group of women with CC and between 6% and 25% in women without CC.

The women with CC presented a specific bacterial community, which could not be isolated in women without CC, consisting of seven species, *Streptococcus urinalis, Escherichia coli, Bacillus safensis, Bacillus malikii, Corynebacterium jeikeium, Corynebacterium striatum*, and *Lactobacillus rhamnosus*. Likewise, the group of women without CC presented eight species not found in those with CC, consisting of *Staphylococcus pasteuri, Staphylococcus auricularis, Staphylococcus capitis* subsp. *capitis*, *Facklamia hominis, Paenibacillus urinalis, Pseudocitrobacter faecalis, Brevibacterium masiliense* and *Klebsiella oxytoca*.

Clinical stages of the disease were divided for analysis according to the presence or absence of parametrial invasion. Stages IB2 to IIA2 (without parametrial invasion) presented 8 bacterial species. The women with parametrial invasion (stages IIB to IVA) presented the 12 species identified. The species with the highest predominance was *Staphylococcus epidermidis*, as it was present in 100% of the patients without parametrial invasion, compared to 44.4% found in women with parametrial invasion; this result is statistically significant (p<0.01) (**Table 3**).

**Table 3:** Difference in proportions of bacterial species found according to the clinical stage of the disease and histological type of tumor.

Identified species	Clinical stage		Significance	Tumor histological type		Significance
	IB2, IIA, IIA1, IIA2 (Without parametrial invasion) n = 15	IIB, IIIA, IIIB, IVA (With parametrial invasion) n = 45		Squamous cell carcinoma = 51	Adenocarcinoma n = 9	
	% (Frequency)	% (Frequency)		% (Frequency)	% (Frequency)
*Staphylococcus epidermidis*	100 (15)	44.4 (20)	<0.01*	56.8 (29)	66.6 (6)	0.72
*Corynebacterium amycolatum*	40.0 (6)	40.0 (18)	1.00	31.3 (16)	88.8 (8)	<0.01*
*Enterococcus faecalis*	26.0 (4)	22.2 (10)	0.73	21.5 (11)	33.3 (3)	0.18
*Escherichia fergusonii*	6.6 (1)	15.5 (7)	0.66	15.6 (8)	0.0 (0)	0.33
*Corynebacterium jeikeium*	6.6 (1)	11.1 (5)	1.00	11.7 (6)	0.0 (0)	0.57
*Streptococcus agalactiae*	0.0 (0)	13.3 (6)	0.32	9.8 (5)	11.1 (1)	1.00
*Bacillus safensis*	13.3 (2)	8.8 (4)	0.63	9.8 (5)	0.0 (0)	1.00
*Lactobacillus rhamnosus*	13.3 (2)	6.6 (3)	0.59	7.8 (4)	11.1 (1)	0.57
*Streptococcus urinalis*	0.0 (0)	8.8 (4)	0.56	3.9 (2)	11.1 (1)	0.39
*Bacillus malikii*	6.6 (1)	6.6 (3)	1.00	3.9 (2)	22.2 (2)	0.10
*Escherichia coli*	0.0 (0)	6.6 (3)	0.56	3.9 (2)	11.1 (1)	0.39
*Corynebacterium striatum*	0.0 (0)	2.2 (1)	1.00	0.0 (0)	11.1 (1)	0.15

%, percentage; *significance level p < 0.05, Fisher’s exact test.

Regarding the species found according to the histological type of tumor, women with squamous cell carcinoma presented 11 of the 12 species identified (91.6%), except for *Corynebacterium striatum*. Women with adenocarcinoma presented 9 of the 12 species (75%). The difference in proportions between both groups for all bacteria only showed statistically significant differences in the presence of *C. amycolatum* (p<0.01), with a higher proportion in women with adenocarcinoma (**Table 3**).

## Discussion

Cervical cancer is one of the leading health problems in the world; there are multiple reports about the risk factors associated with HPV infection, its persistence, and cancer development. We have detected four risk factors in the Mexican population analyzed in this study. Education level, which can be related to socioeconomic status, was lower in the group of women with cervical cancer, coinciding with previous studies showing that detection of the disease is later as schooling level decreases (Chen et al., 2012; Thulaseedharan et al., 2012; Akinlotan et al., 2017; Nessa et al., 2020). The onset of active sexual life was at an earlier age in women with CC, and it has been reported that early age at first intercourse may predispose women to HPV infection (Creatsas and Deligeoroglou, 2012; Dahiya et al., 2017). Parity, which was higher in women with CC. This risk factor may be related to hormonal changes and epithelial modifications caused by repeated cervical trauma (Thulaseedharan et al., 2012; Nessa et al., 2020). And finally, the use of contraceptive methods, in which the higher use of hormonal methods by women with CC is observed.

It has been commonly reported that the composition of “healthy” or “normal” cervicovaginal bacterial communities is based on the presence or absence of lactobacilli. Ravel et al. (2011) have classified it into multiple categories or “clusters” (I-V) based on the dominant *Lactobacillus* species and the ethnic group in which they are found. Bacterial communities I, II III and V, are dominated by *Lactobacillus crispatus*, *L. gasseri*, *L. iners*, and *L. jensenii*, respectively, and were found more frequently in Asian and white women. Bacterial community IV is not dominated by any lactobacillus species and has a high presence of anaerobic bacteria. This group was overrepresented in Hispanic and black women, concluding that vaginal bacterial communities not dominated by *Lactobacillus* species are common and appear normal in these ethnic groups (Ravel et al., 2011; Kroon et al., 2018). However, according to Martínez-Peña et al. (Martínez-Peña et al., 2013), the healthy cervicovaginal microbiota of Mexican women is mainly composed of *L. acidophilus* group lactobacilli, namely *L. gasseri*, *L. fermentum*, *L. rhamnosus*, *L. jensenii*, *L. crispatus* and *L. brevis*, which is in contrast to that described by Ravel et al. (Ravel et al., 2011). Likewise, our results also show the presence of critical aerobic bacteria in women without CC. We believe that further studies are needed to establish the normal parameters of this bacterial community and to determine with greater certainty the variation according to population, genotype, or the presence of disease.

Several studies have already exposed the link of cervicovaginal bacterial microbiota and CC during the natural history of the disease, where it is shown that from HPV infection and up to the presence of a cervical intraepithelial lesion, there is an association between cervical dysbiosis and disease (Mitra et al., 2015; Usyk et al., 2020; Onywera et al., 2021). The most studied entity is bacterial vaginosis, in which a higher diversity of strict anaerobic species and a considerable decrease in lactobacilli are observed (Sodhani et al., 2017; Brusselaers et al., 2019; Kovachev, 2020). However, current reports suggest that aerobic bacteria may also play an essential role in this pathology (Vieira-Baptista et al., 2016; Plisko et al., 2021).

This study aimed to detect aerobic bacteria related to the presence of CC. Our findings show that most isolates corresponded with facultative aerobic bacteria, which may demonstrate their high prevalence both in the bacterial communities present in women with CC and those without the pathology. According to Donders et al. (2017), one of the essential elements in aerobic vaginitis is colonization for *Escherichia coli, Staphylococcus aureus, S. epidermidis, Streptococcus agalactiae*, and *Enterococcus faecalis*. In our study, both groups presented bacteria typical of aerobic vaginitis. However, there were more strains isolated in women with cancer. This finding could contribute to the observation of Plisko et al. (Plisko et al., 2021), who concluded that changes in the microbiota associated with aerobic vaginitis are related to pre-invasive lesions. With our results, we can consider that these changes may also be related to cancer establishment.

Regarding the presence of this same group of bacteria in women without CC, although with lower strains isolated and fewer species, we can elucidate that this may represent infection or dysbiosis, but HPV infection is probably necessary for neoplastic changes to develop. According to Vieira-Baptista et al. (Vieira-Baptista et al., 2016), aerobic vaginitis is not associated with an increased risk of acquiring HPV, but it has been associated with the persistence of infection. It can therefore be considered a risk factor. The consequences of this and why it may or may not be related to the development of neoplasia should be studied under future designs and should also be studied considering the presence or not of HPV infection. These are two aspects that we did not consider in our study, representing points for improvement in subsequent projects.

Concerning the clinical stage of the disease, we found that more advanced stages presented a higher number of isolates and a more significant number of species coincident with aerobic vaginitis. On the contrary, less extended clinical stages showed a lower number of species and a more consistent bacterial community, with a better local ecology; this is consistent with previous studies (Audirac-Chalifour et al., 2016; García-García et al., 2017).

About the presence of these species and the severity of the disease, we know that aerobic vaginitis is an entity that by itself generates discomfort by producing a variable amount of inflammation and thinning of the vaginal epithelium (Oerlemans et al., 2020). We can elucidate that this dysbiotic microbiota could drive the pathology by promoting immune evasion, favoring tumor cell survival. It could also act by producing an inflammatory environment that in the long term promotes tissue damage resulting in an ecosystem more conducive to disease aggravation. These two aspects have been previously addressed to relate carcinogenesis to aerobic vaginosis, but not to explain the severity of the disease (Vieira-Baptista et al., 2016; Donders et al., 2017; Plisko et al., 2021). We suggest studies whose aim is directed to the analysis of these variables.

No marked differences were observed in histological type for the microbiota associated with either of the two carcinomas found in this study group. In general, squamous cell carcinoma was related to a more significant bacterial presence, both in quantity and species number; this could be influenced by the anatomical characteristics of the tumor, which could provide a more favorable habitat for the installation of these species. A larger sample size regarding adenocarcinoma is likely to be necessary to obtain conclusive results.

Our study does not provide fundamentals to determine a causal association between the bacteria characteristic of aerobic vaginitis and cervicovaginal neoplasia; we do not have elements to clarify if aerobic vaginitis promotes carcinogenesis or if it is established as a consequence of cancer development due to the deprivation of the immune system when trying to counteract the neoplasia. But considering previous studies that have demonstrated a link between an altered vaginal microbiome and pre-invasive cervical disease, we propose that aerobic vaginitis and the inflammation with which it is accompanied are probably crucial for the progression of preneoplastic lesions to cancer (Vieira-Baptista et al., 2016; Plisko et al., 2021).

This cross-sectional study, developed as a preliminary experiment for further research, made it possible to explore the essential characteristics of the cervicovaginal microbiota associated with cervical cancer, the differences between women with and without the pathology, and the need for more in-depth studies. It also allowed us to establish variables for future analyses, funding needs, and sample collection and treatment methods.

Although the “omics” era has accelerated all aspects of biological research, it is advisable to integrate traditional microbiology methods to formulate optimal study designs. Conducting a research project depends primarily on the scientific objective and the availability of samples, the quality of the samples, and the cost of the experiment. Massive sequencing of 16S *r*RNA gene amplicons and metagenomic sequencing is used to obtain a broader view of bacterial communities and determine potential functions and genome recovery. However, it generally involves the development of large-scale research projects with sufficient funding. In developing countries, where financial resources in science and technology are scarce, it is essential to perform detailed designs that properly manage samples, variables, and financial resources, minimizing losses. Our preliminary identification of the microbiota as a preliminary approach to metagenomic studies can contribute to establishing this type of design.

We can conclude that we were able to establish the presence of bacteria typical of the entity known as aerobic vaginitis in women with CC and that it is crucial to carry out more in-depth studies. There is little information about this dysbiosis and its association with cervical cancer, so the reported findings are helpful to corroborate that there are bacterial communities that, although they may be abundant, so far have been little studied and whose influence on the establishment, development, or cure of CC are unknown. Our report shows that cross-sectional study designs and traditional microbiological methodology are helpful to determine whether there is a need for more complex studies and to know whether it is necessary to optimize the management of variables, samples, and financial resources.

Among the limitations of this study, we consider it necessary to report that we did not manage to extend the participants’ last use of antimicrobials and antifungals, so we limited ourselves to 30 days as exclusion criteria. We know that this period is short for the microbiota to recover satisfactorily and return to “normal”. However, we tried to lessen its impact by choosing a large sample size that balanced some of the participants’ confounding results. Subsequent studies should address this issue. Likewise, we could not diagnose aerobic vaginitis or identify HPV; however, this is a precedent for future work to determine the association between aerobic vaginitis and HPV infection or persistence. Finally, we consider it a limitation to have performed a cross-sectional study. However, this is an antecedent for future studies to address longitudinal designs in which the relationship between aerobic vaginitis and cervical cancer can be evaluated more effectively.

## Data Availability Statement

The obtained sequences are publicly available in the GenBank of the National Center for Biotechnology Information with access numbers MH108987-MH109026 (Group 1) and MH158254-MH158283 (Group 2). All other data in the study are available on request from the corresponding author.

## Ethics Statement

The studies involving human participants were reviewed and approved by Ethics and Research Committee of the National Cancer Institute of Mexico (INCan, 016/11/ICI-CEI/1016). The patients/participants provided their written informed consent to participate in this study.

## Author Contributions

GM-L, JC-M, MR-M, LB-M, HS-T, and NR-D participated in the study design. JC-M, MR-M, and IR-C collected epidemiological data. GM-L, MR-M, and IR-C performed sample collection. GM-L, HS-T, and NR-D executed the experiment. GM-L and LB-M performed the analysis of results. All authors contributed to the article and approved the version presented.

## Conflict of Interest

The authors declare that the research was conducted in the absence of any commercial or financial relationships that could be construed as a potential conflict of interest.

## Publisher’s Note

All claims expressed in this article are solely those of the authors and do not necessarily represent those of their affiliated organizations, or those of the publisher, the editors and the reviewers. Any product that may be evaluated in this article, or claim that may be made by its manufacturer, is not guaranteed or endorsed by the publisher.
